# Comparative effectiveness and safety of laser, needle, and “quick fenestrater” in *in situ* fenestration during thoracic endovascular aortic repair

**DOI:** 10.3389/fcvm.2023.1250177

**Published:** 2023-09-29

**Authors:** Xiaokai Wang, Jianjin Wu, Kangkang Zhi, Sili Zou, Jie Jin, Jun Bai, Lefeng Qu

**Affiliations:** Department of Vascular and Endovascular Surgery, Second Affiliated Hospital of Naval Medical University, Shanghai, China

**Keywords:** aortic arch, aortic disease, *in situ* fenestration, thoracic endovascular aortic repair (TEVAR), quick fenestrater (QF)

## Abstract

**Background:**

Special instruments are needed for the revascularization of aortic branches in *in situ* fenestration during thoracic endovascular aortic repair (TEVAR). This prospective study compared the effectiveness and safety of three currently used fenestraters: laser, needle, and Quick Fenestrater (QF).

**Methods:**

In all, 101 patients who underwent TEVAR for aortic disease (dissection, *n* = 62; aneurysm, *n* = 16, or ulcer, *n* = 23) were enrolled. All patients were randomly assigned to three groups: 34 were assigned to laser fenestration, 36 to needle fenestration, and 31 to QF fenestration. The epidemiological data, treatment, imaging findings, and follow-up outcomes were analyzed using data from the medical records.

**Results:**

The technical success rates of the laser, needle, and QF fenestration groups were 94.1%, 94.4%, and 100% (*p* > 0.05). After correction of mixed factors such as age and gender, it was showed the average operative time (Laser group: 130.01 ± 9.36 min/ Needle group: 149.80 ± 10.18 min vs. QF group: 101.10 ± 6.75 min, *p* < 0.001), fluoroscopy time (Laser group: 30.16 ± 9.81 min/ Needle group: 40.20 ± 9.91 min vs. QF group: 19.91 ± 5.42 min, *p* < 0.001), fenestration time (Laser group 5.50 ± 3.10 min / Needle group 3.50 ± 1.50 min vs. QF group 0.67 ± 0.06 min, *p* < 0.001), and guide wire passage time after fenestration (Laser group 5.10 ± 1.70 min / Needle group 4.28 ± 1.60 min vs. QF group 0.07 ± 0.01 min, *p* < 0.001) were all shorter with QF fenestration than with the other two tools. The overall perioperative complication rates of the laser, needle, and QF fenestration groups were 5.9%, 5.6%, and 0% (p > 0.05): One case of sheath thermal injury and one case of vertebral artery ischemia occurred in the laser fenestration group; one case each of access site hematoma and brachial artery thrombosis were reported in the needle fenestration group. 89 (88.1%, 89/101) patients were followed for a median of 12.6 ± 1.6 months. The overall postoperative complication rates of the laser, needle, and QF fenestration groups were 3.3%, 6.5%, and 0% (p > 0.05): In the laser fenestration group, there was one death due to postoperative ST-segment elevation myocardial infarction; in the needle fenestration group, one patient developed occlusion of the bridge stent; no complications occurred in the QF group.

**Conclusion:**

All three fenestration methods were effective in reconstructing supra-arch artery during TEVAR. QF fenestration required less contrast agent, with a shorter surgery duration and fewer complications than laser and needle fenestration.

## 1. Introduction

Aortic aneurysm, aortic ulcer, and aortic dissection are the major thoracic aortic diseases. These diseases greatly affect public health (1, 2). Thoracic endovascular aortic repair (TEVAR) is an effective method for the treatment of such diseases. However, aortic diseases involving supra arch branches are considered contraindications to TEVAR due to the retention of the supra arch branches (3–5). In situ fenestration can extend the sealing zone during TEVAR and has shown the potential for the revascularization of aortic branches (6–8). Three instruments are generally used in *in situ* fenestration during TEVAR—laser, needle, and “Quick Fenestrater” (QF) (9). In 2004, Mc Williams et al. (10) reported satisfactory clinical results with reconstruction of the left subclavian artery by *in situ* fenestration with a reversed end of a 0.018-inch guidewire. In 2009, Murphy et al. (11) first applied laser fenestration technology during TEVAR to preserve the left subclavian artery. There are increasing applications of *in situ* fenestration technology, and it is considered the most consistent technology with human anatomy and hemodynamics and is a safe and effective method (12, 13).

**Figure 1:**
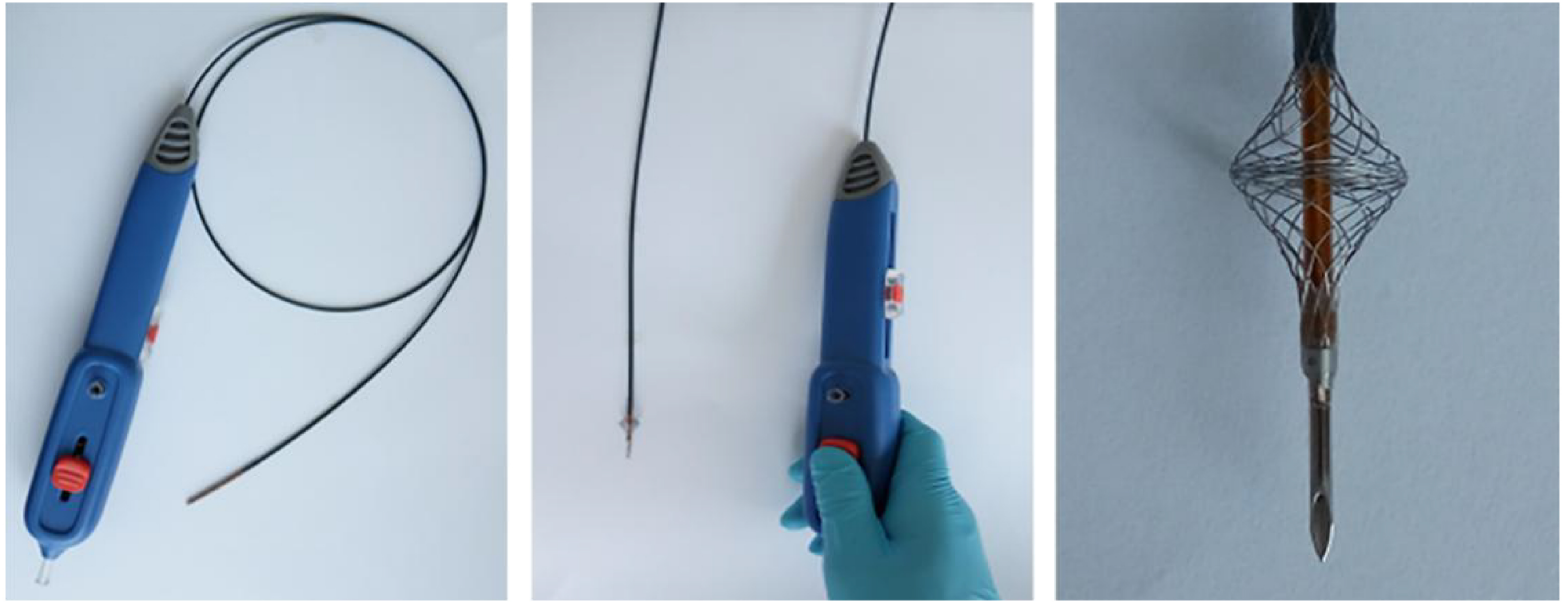
The structure and working principle of quick fenestrater (QF).

Both needle and laser *in situ* fenestration have limitations, such as injury to the contralateral aortic wall, long fenestration time, thermal injury, and invisible head end of the guide wire of laser fenestration (14). Therefore, our team pioneered a device, “Quick Fenestrater” (QF) specifically used for *in situ* fenestration of supra aortic vessels (Figure 1) (15). In our study, we showed that QF was a safe and effective method, but we did not compare the advantages and disadvantages of the three fenestration methods. In this study, we compared the effectiveness and safety of laser, needle, and QF *in situ* fenestration during the perioperative period and 1-year follow-up.

## 2. Methods

### 2.1. Patient enrollment

In this prospective cohort study, 101 patients were enrolled from December 2016 to October 2019 at the Department of Vascular Surgery in Shanghai Changzheng Hospital. The inclusion criteria were (1) age above 18 years, (2) at least 1 supra-aortic branch encroached by thoracic lesions, (3) landing zone not long enough (≤15 mm) for fixation of the aortic endograft so coverage of the LSA or LCCA had to be performed, (4) aortic disease confirmed by at least 1 radiologic examination (e.g., computed tomography angiography [CTA], magnetic resonance angiography [MRA]), (5) patent supra-aortic branch, and (6) type I/II aortic arch. The exclusion criteria were (1) cardiopulmonary and renal insufficiency contraindicating general anesthesia (according to the anesthesiologist), (2) severe infection causing high fever or organ dysfunction, (3) allergy to contrast medium, (4) adverse cardiovascular or cerebrovascular event within 3 months before intervention, (5) occlusion or stenosis of the supra-aortic branch or severe twisting of the arteries to be fenestrated, (6) no appropriate peripheral access, (7) Stanford A aortic lesions, (8) type III aortic arch and (9) Marfan syndrome. The protocol for this study was approved by the participating hospital’s Ethics Committee, and informed consent was obtained from each participant.

### 2.2. Surgical procedure

The enrolled patients underwent *in situ* laser, needle, or QF fenestration according to their order of admission. All interventions were performed by advanced interventional radiologists. The procedures were performed under general anesthesia. Two ProGlide vascular sutures (Perclose ProGlide, Abbott Inc, USA) were preset for right femoral artery puncture, and a 12-F sheath was inserted. A 7-F sheath (pre-left carotid artery fenestration) was punctured and inserted via the left common carotid artery, and a 6-F 55 cm renal artery sheath (Flexor® Check-Flo® Introducer, Cook, Inc, USA) was punctured and inserted into the left brachial artery. A 5-F labeled pig-tail catheter was introduced into the sheath of the right femoral artery to the ascending aorta, and the aortic size was measured. A Lunderquist super-stiff guide wire was inserted, and the pigtail catheter was withdrawn to the ascending aorta through the left brachial artery or carotid artery sheath for real-time intraoperative angiography. After angiographic evaluation of the aortic lesions, aortic stents of appropriate size (Capitivia, Medtronic, USA/Ankura, Lifetech, China) were introduced along the super stiff guide wire, accurately positioned, and released to repair thoracic aortic lesions and cover the supra arch branch arteries. Advanced through the 0.035 inch support catheter, the needle (reversed end of a 0.018 inch guidewire) or intravenous laser catheter (LFK-SLT30, Rafcon, China) or QF (Suzhou Innomed Medical Device, China) was introduced along the supra arch branch artery and guided to the branch artery for stent graft fenestration. After fenestration, the needle, intravenous laser catheter, or QF was advanced into the aortic lumen. The fenestration instruments were withdrawn, and another angiogram was made to confirm that the needle, intravenous laser catheter, or QF was positioned in the aortic cavity, and a 0.035 inch guide wire was introduced along the catheter to the ascending or descending aorta. A balloon (Mustang, Boston Scientific Corporation, USA) of appropriate size to expand the fenestration was positioned. The stent deployed in the super arch artery had a 5 mm protrusion in the aortic arch, and the distal end in left subclavian artery did not cover the orifice of the vertebral artery. Post dilatation was carried out if necessary. Finally, aortography was performed to check that the intervention was carried out successfully.

### 2.3. Outcomes

After the intervention, follow-up was conducted at 1 month, 6 months, and annually thereafter. The following perioperative outcomes were assessed: success rate of fenestration; average durations of intervention, fluoroscopy, rupture, and guide wire passage after fenestration (from guide wire touching the membrane to passing through the hole); and amount of contrast agent used. Follow-up outcomes included 30-day mortality, type I and III endoleaks, retrograde dissection, mortality, stent patency rate, stent morphology, secondary re-interventions, and severe cardiovascular and cerebrovascular events. Computed tomographic angiography of the aorta was performed to evaluate the rates of branch stenting, internal leakage rate, and secondary intervention.

### 2.4. Statistical analysis

The data were analyzed with SPSS 22.0 software. The data were expressed as mean values ± SD. Continuous variables were presented as median and range in the case of nonparametric distributions, and comparisons were made using the Mann-Whitney test. Continuous variables are presented as means ± standard deviations in cases of parametric distributions, and comparisons were made using the independent t-test. Categorical variables were compared using the Chi-square test or Fisher’s exact test and reported as frequencies (percentages). A *p* value of <0.05 was considered statistically signiﬁcant.

## 3. Results

### 3.1. Patient demographics and presentation

In all, 101 patients underwent *in situ* fenestration during TEVAR between May 2016 and October 2019 (85 males and 16 females; mean age, 67 ± 10 years; 71 acute onset). In all, 34 patients underwent 40 *in situ* laser fenestrations during TEVAR (aortic dissection: *n* = 21, aortic aneurysm: *n* = 5, aortic ulcer: *n* = 8); 36 patients underwent 43 *in situ* needle fenestrations during TEVAR (aortic dissection: *n* = 22, aortic aneurysm: *n* = 6, aortic ulcer: *n* = 8); and 31 patients underwent 37 *in situ* QF fenestrations during TEVAR (aortic dissection: *n* = 19, aortic aneurysm: *n* = 5, aortic ulcer: *n* = 7). The baseline characteristics, including risk factors and comorbidities, were not significantly different among the three groups (Table 1).

**Table 1:** Baseline characteristics of patients.

	Laser fenestration	Needle fenestration	QF fenestration	*P*
	*N* = 34	*N* = 36	*N* = 31	
Average age	65.7	67.2	68.1	
Male	29 (85.3%)	30 (83.3%)	26 (83.9%)	0.974
Acute onset	24 (70.6%)	25 (69.4%)	22 (71%)	0.990
Disease classification	** **			
Thoracic aortic dissection	21 (61.8%)	22 (61.1%)	19 (61.3%)	0.998
Thoracic aortic aneurysm	5 (14.7%)	6 (16.7%)	5 (16.1%)	0.974
Thoracic aortic ulcer	8 (23.5%)	8 (22.2%)	7 (22.6%)	0.991
Risk factors	** **			
Hypertension	16 (47.1%)	17 (47.2%)	14 (45.2%)	0.983
Hyperlipidemia	10 (29.4%)	11 (30.6%)	10 (32.3%)	0.969
Diabetes mellitus	5 (14.7%)	6 (16.7%)	5 (16.1%)	0.974
Smoking	26 (76.5%)	28 (77.8%)	24 (77.4%)	0.991
Comorbidities	** **			
Severe COPD	3 (8%)	5 (13.8%)	4 (12.9%)	0.800
Cardiac dysfunction	10 (29.4%)	8 (22.2%)	12 (38.7%)	0.338
Hepatic insufﬁciency	0	1 (2.7%)	0	1.000
Renal insufﬁciency	5 (14.7%)	7 (19.4%)	5 (16.2%)	0.862

### 3.2. Operative data

**Figure 2:**
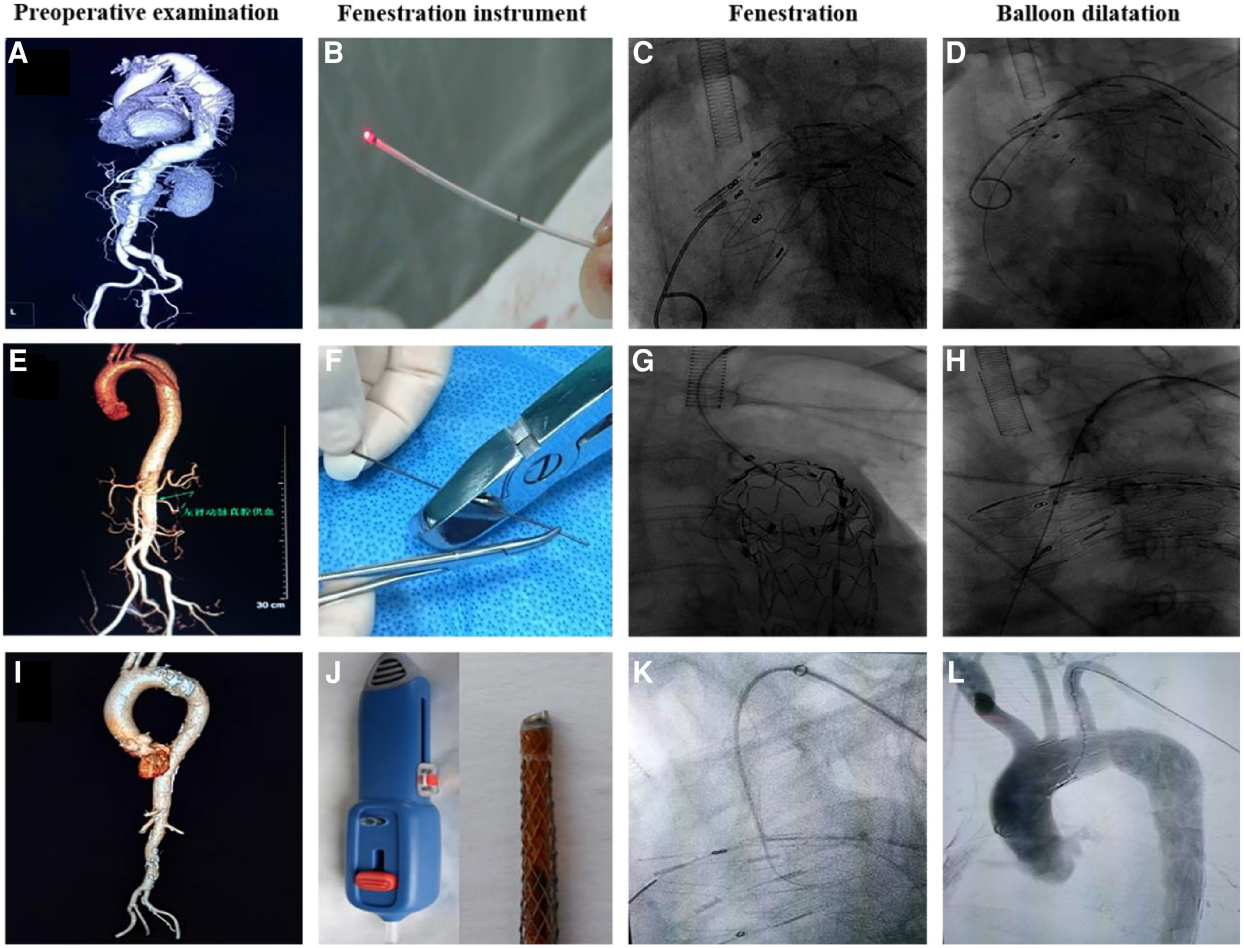
Preoperative examination, fenestration instrument, fenestration, and balloon dilatation using three different fenestration methods. (**A**–**D**) Laser fenestration, (**E**–**H**) needle fenestration, (**I**–**L**) QF fenestration.

TEVAR was technically successful in 94 patients (Figure 2). The technical success rates of the laser, needle, and QF fenestration groups were 94.1%, 94.4%, and 100%, respectively, showing an insignificant difference (*p* > 0.05). In the *in situ* laser fenestration group, one procedure failed due to an awkward fenestration angle of the laser guide wire. In the *in situ* needle fenestration group, one procedure failed because the needle had failed to puncture the membrane. After correction of mixed factors such as age and gender, variance analysis and Kruskal-Wallis test results showed the average operation, fluoroscopy, fenestration, and guide wire passage durations after fenestration were shorter in the QF fenestration group (*p* < 0.001). Moreover, the average amount of contrast agent in the QF fenestration group was less than that in the needle fenestration and laser fenestration group (*p* < 0.001) (Table 2).

**Table 2:** Perioperative data for the three cohorts.

	Laser fenestration	Needle fenestration	QF fenestration	*P*
Branch artery revascularized	** **	** **	** **	** **
LSA	28 (28/34)	29 (29/36)	25 (25/31)	
LSA + LCCA	6 (6/34)	7 (7/36)	6 (6/31)	
Technical success rate	94.1%	94.4%	100.0%	0.545
Intraoperative vascular injury	2	2	0	0.545
Operation process	** **			
Average operation time (min)	130.01 ± 9.36	149.80 ± 10.18	101.10 ± 6.75	<0.001
Average fluoroscopy time (min)	30.16 ± 9.81	40.20 ± 9.91	19.91 ± 5.42	<0.001
Average fenestration time (min)	5.50 ± 3.10	3.50 ± 1.50	0.67 ± 0.06	<0.001
Average contrast material (ml)	152.02 ± 30.12	180.21 ± 20.23	100.01 ± 15.15	<0.001
Average guide wire passage time after fenestration (min)	5.10 ± 1.7	4.28 ± 1.6	0.07 ± 0.01	<0.001
Patency rate of branch stent	100%	100%	100%	

LSA, Left subclavian artery; LCCA, Left common carotid artery.

All the *in situ* fenestrated arteries were found to be patent on postoperative follow-up CTA imaging and clinical symptoms.

### 3.3. Clinical follow-up

**Figure 3:**
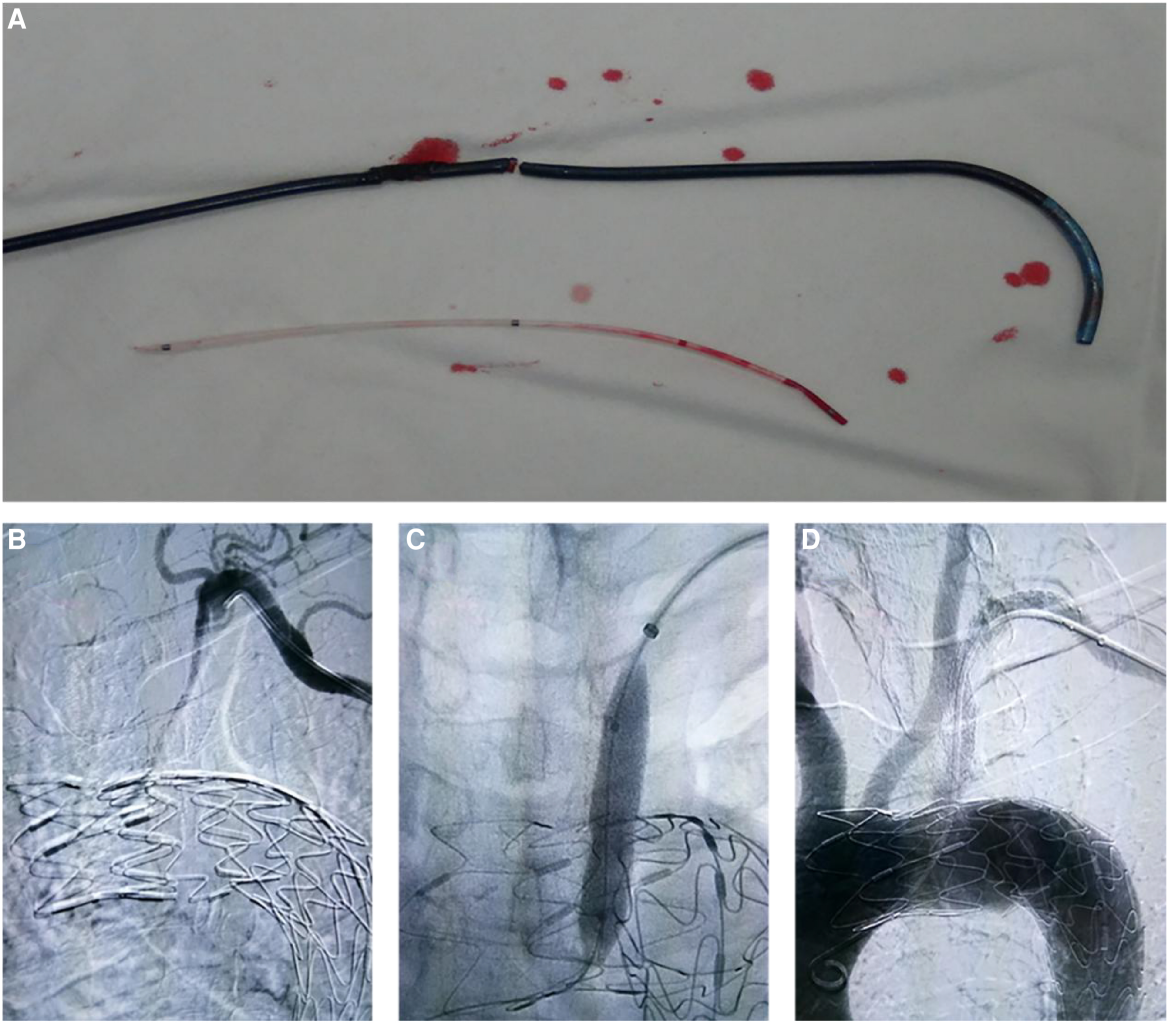
Complications in laser and needle fenestration groups. (**A**) In the laser fenestration group, one patient sustained sheath injury caused by laser ablation. (**B**–**D**) In the needle fenestration group, one patient developed subclavian artery occlusion after the intervention, but it was recanalized.

The overall Perioperative complication rates of the laser, needle, and QF fenestration groups were 5.9%, 5,6%, and 0% (*p* > 0.05). In the perioperative period, no cerebral infarction, myocardial infarction, transient ischemic attacks, or other neurologic complications occurred. In the *in situ* laser fenestration group, one patient had a sheath injury due to laser heat (Figure 3A), and one patient presented with intraoperative vertebral artery ischemia. In the *in situ* needle fenestration group, one patient presented with an access-site hematoma, and one patient presented with a femoral arterial thrombus. No endoleak (include type I and type III), false lumen thrombosis, or subsequent remodeling of the aorta was evident.

**Figure 4:**
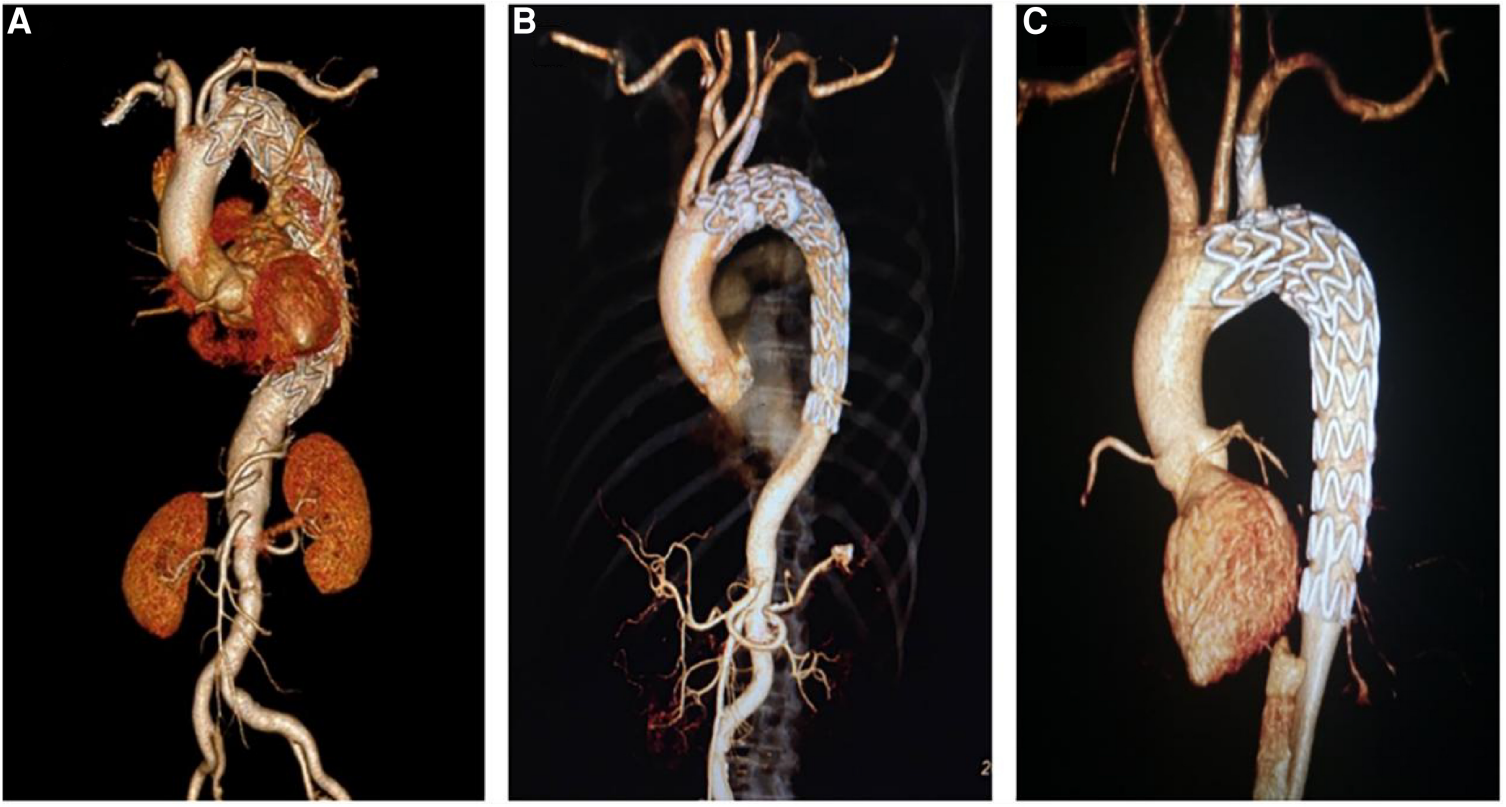
Postoperative computed tomography angiography of three different fenestration methods. (**A**) Laser fenestration, (**B**) needle fenestration, (**C**) quick fenestration.

A total of 89 (88.1%, 89/101) patients were followed for a median of 12.6 ± 1.6 months. At the 1-year follow-up, the overall postoperative complication rates of the laser, needle, and QF fenestration groups were 3.3%, 6.5%, and 0% (p > 0.05). In the laser fenestration group, there was one death due to postoperative ST-segment elevation myocardial infarction, with no direct association with the intervention. In the needle fenestration group, one patient developed occlusion of the bridge stent at 3-month follow-up. The occlusion was recanalized after intervention (Figure 3B–D). There were no complications in the QF group. No reverse tear or endoleak (type I and type III) was observed in any of the three groups (Table 3, Figure 4).

**Table 3:** Follow-up of patients.

	Laser fenestration *N* = 30/34	Needle fenestration *N* = 31/36	QF fenestration *N* = 28/31
Mean follow-up time (months)	12.7 ± 1.5	13.0 ± 1.4	12.1 ± 1.7
30-day mortality	0	0	0
Type I endoleak	0	0	0
Type III endoleak	0	0	0
Retrograde dissection	0	0	0
Follow-up mortality	1	0	0
Stent compression	0	1	0
Secondary re-interventions	0	1	0

## 4. Discussion

The fenestration methods used in TEVAR include mechanical fenestration, represented by self-made guide wires or puncture needles, and thermal fenestration, represented by laser/radio frequency. Our center developed a special fenestration instrument, the “Quick Fenestrater” (QF), which has demonstrated safety and efficacy in a previous single-center clinical study (15). On analyzing the data of the QF, laser, and needle fenestration groups, we found that (1) all three methods were effective for *in situ* fenestration of the superior arch artery, each with a technical success rate of over 90%; the QF fenestration rate was 100% and (2) while there was no significant difference in the average durations of operation, fluoroscopy, fenestration, and guide wire passage after fenestration and volume of contrast agent used between the needle and laser fenestration groups, the lowest measurements were observed in the QF group (*P* < 0.001). (3) There were no significant differences in the endoleak, postoperative all-cause mortality, secondary surgical intervention, and branch stent patency rates among the three groups during follow-up.

In situ fenestration during TEVAR is a safe and effective method for endovascular reconstruction of the branch arteries above the aortic arch (16–19). In 2016, in a meta-analysis of 118 articles conducted by Crawford et al., the success rate of *in situ* fenestration was 96%, and the rate of comprehensive complications (death, stroke, and paraplegia) was 7% (20). In situ fenestration has the advantages of rapid fenestration, repeatability, minimally invasiveness, and few complications.

Nevertheless, during the study of fenestration instruments, we found some undesirable features: needle fenestration requires manual or other types of puncture needles (e.g., biopsy needles); puncture needles have high rigidity and poor flexibility and cannot pass through the twisted subclavian artery, resulting in a relatively long fenestration time (21). Moreover, the direction of the puncture tip is difficult to adjust, and there is a risk of damaging the arterial intima and puncturing the opposite vessel wall (22–24). In this study, in the needle fenestration group, one patient developed an occlusion on the bridging stent at the 3-month follow-up; perhaps, because of the large torsion angle of the subclavian artery, the intima was damaged by the needle, resulting in a dissection. During recanalization, angiography showed that the stent itself was in good shape, and the dissection was seen at the tortuous subclavian artery at the distal end of the stent; the arterial dissection was repaired by balloon expansion and implantation of a bare stent.

Laser *in situ* fenestration is invisible under fluoroscopy at the head end, and the direction of the head end is difficult to adjust, resulting in the risk of thermal damage (25, 26). In the laser group, thermal injury of sheath due to the supporting catheter damaged the intima of the subclavian artery and caused thrombosis. After laser fenestration, the laser fiber must be pushed out and the guide wire must be exchanged; in this process, because the fenestration window is small and invisible, exchanging the guide wire often takes time; if and when it is difficult to pass through, the membrane must be ruptured with laser again to enlarge the window (27, 28). In the laser fenestration group, one patient, treated with local anesthesia, had sudden transient ischemia of the vertebral artery, and the symptoms resolved after the fenestration was completed. According to previous reports, the laser could lead to the formation of air bubbles, and the bubbles could enter cerebral blood vessels and cause a stroke, which could have occurred in our case (29).

In contrast to other fenestraters, QF can fix the access point in the central area of the blood vessel through the support bracket at the front end and ensure the fenestration is relatively parallel to the vessel wall. QF uses a needle with adjustable strength and depth control to quickly fenestrate, thus preventing vascular damage. After successful fenestration, the guide wire, preset from the needle lumen, can quickly enter the aortic lumen to complete the fenestration process. The whole process is simple, smooth, and time saving. In summary, QF has significant advantages over the other two fenestration methods.

The shape of the aortic arch and the angle of the branch blood vessels affect the success rate of the fenestration. Theoretically, fenestration perpendicular to the membrane has the highest success rate. For laser fenestration, the laser head must be placed on the stent graft. Given the arch type and angle of branch vessels, the laser head may slip off, resulting in failure of the fenestration. Needle fenestration faces the same problem. Moreover, the head ends of the needle and laser fibers are uncontrollable and their angles cannot be changed (24, 30). Because of these disadvantages of needle and laser fenestration, we developed the QF to fix the access point in the central area of the blood vessel through the support bracket at the front and use the membrane-perforating needle with adjustable strength and controllable depth to quickly fenestrate and minimize the risk of vascular damage. By optimizing the operation and simplifying the operation steps, the success rate of fenestration is increased, and the operation time is reduced. In the present comparison of QF, needle, and laser fenestration, we found that by reducing the intervention time, QF fenestration can reduce the amount of contrast agent used and the operator’s radiation exposure, so that complications in high-risk patients, such as renal insufficiency, are reduced.

## 5. Conclusions

Our comparative study of the effectiveness of the recently designed QF, needle, and laser as fenestration tools in endovascular aortic arch repair showed that the QF was associated with a shorter surgical duration, lower volume of contrast agent used, and better safety. At the follow-up, there were no complications in the patients who underwent endovascular aortic arch repair with the QF.

## Data Availability

The original contributions presented in the study are included in the article/Supplementary Material, further inquiries can be directed to the corresponding authors.
